# The striking divergence of ABCB1 mRNA expression and P-glycoprotein protein levels in M1 and M2 macrophages associates with the microRNA miR-21-3p, a regulator of “RNA binding protein, mRNA processing factor”

**DOI:** 10.1007/s00210-026-05288-8

**Published:** 2026-04-11

**Authors:** Katharina Hamburg, Johanna Weiss, Julia Carolin Stingl, Dirk Theile

**Affiliations:** Internal Medicine IX-Department of Clinical Pharmacology and Pharmacoepidemiology, Heidelberg University, Medical Faculty of Heidelberg, Im Neuenheimer Feld 410, 69120 Heidelberg, Germany

**Keywords:** Macrophages, Drug transporters, P-glycoprotein, MicroRNA, MiR-21-3p

## Abstract

Macrophages are important hosts for intracellular pathogens. Drug transporters modulate the uptake and thus efficacy of antibiotics in macrophages. However, there is only few data comparing drug transporter expression levels in polarized pro-inflammatory M1 to anti-inflammatory M2 macrophages. THP-1 monocytes were differentiated with 200 nM phorbol 12-myristate 13-acetate for 72 h and subsequently polarized to the M1 (50 ng/mL lipopolysaccharide, 20 ng/mL interferon-gamma; 48 h) or the M2 (20 ng/mL interleukin-4, interleukin-13; 48 h) phenotype. Using a quantitative polymerase-chain reaction array, mRNA levels of 84 drug transporters were evaluated. Western blotting was used to assess P-glycoprotein (P-gp, encoded by ABCB1) protein expression. Finally, expression of 46 P-gp-regulating microRNA species was evaluated as well. Compared to M2 macrophages, seven transporter genes were lower expressed in M1 cells (e.g., ABCG2 threefold; SLCO2B1 7.5-fold) and 21 genes were higher expressed. ABCB1 mRNA was in fact up-regulated threefold, but 2.4-fold lower at the protein level. This discrepancy associated with a 5.5-fold higher expression of miR-21-3p and a 128-fold lower expression of miR-145-3p. In conclusion, M1 and M2 macrophages differ by drug transporter transcriptomics. The discrepancy of ABCB1 mRNA and P-gp protein levels in M1 macrophages associates with the high abundance of miR-21-3p, a regulator of “RNA binding protein, mRNA processing factor.”

**Supplementary Information:**

The online version contains supplementary material available at 10.1007/s00210-026-05288-8.

## Introduction

Polarized macrophages play an important role during bacterial infections, especially with intracellular pathogens. For instance, while M1 macrophages promote inflammatory responses of the host and mediate strong antimicrobial activity by producing reactive oxygen species or nitric oxide (NO), they are the preferred niche of some bacterial pathogens such as *Listeria monocytogenes* or *Orientia tsutsugamushi* (Thiriot et al. 2020). On the other hand, M2 macrophages have anti-inflammatory functions (e.g., by producing arginase that traps L-arginine to prevent NO synthesis) and promote fibrosis and wound healing through secreting diverse growth factors. However, some bacteria preferentially infect M2 macrophages or their growth is well promoted by an M2-like polarization state. Such M2-targeting pathogens include *Salmonella typhimurium*, *Coxiella burnetii*, or *Chlamydia pneumoniae* (Thiriot et al. 2020). Lastly, polarized macrophages are crucial to the infection with *Mycobacterium tuberculosis* (Mtb) and the related tuberculosis disease. In detail, M2-dominant granulomas are beneficial for the survival and proliferation of Mtb, due to providing immune tolerance or escape. Additionally, Mtb granulomas with enhanced M2/M1-macrophage ratio exhibit enhanced multidrug resistance (Cho et al. 2020).

Given the relevance of polarized macrophages for intracellular bacteria, they are important target cells for antibiotics. To ensure efficacy, sufficient drug levels in the infected macrophages must be achieved. However, macrophage drug accumulation can be modulated by cell membrane-located drug transporters. Indeed, many lines of evidence demonstrate that M1 and M2 cells express a variety of drug transporters that affect drug uptake and thus efficacy, e.g., breast cancer resistance protein (BCRP, coded by *ABCG2)* or multidrug resistance-related protein 1 (MRP1, coded by *ABCC1;* He et al. 2018*)*.

Recently, profound expressional changes of many drug transporters were observed during the monocyte-to-macrophage transition (Hamburg et al. 2026). For instance, M1 polarization enhanced 48 drug transporter genes including a 166-fold up-regulation of *ABCB1*. In contrast, the M2 phenotype had 20 transporter genes up-regulated (e.g., 37-fold enhancement of *ABCG2*) (Hamburg et al. 2026).

However, no direct M1–to–M2 comparison has been reported so far. In this study, THP-1 monocytes were therefore differentiated and polarized to M1 and M2 cells, and their “transportome” was compared at the mRNA level. Given the strikingly divergence of *ABCB1* mRNA and P-gp protein levels in M1 macrophages, expression levels of potential P-gp-regulating microRNA species were recorded as well.

## Materials and methods

### Materials

THP-1 cells were obtained from the American Type Culture Collection (ATCC, Manassas, USA) and RPMI 1640 medium was purchased from PanBiotech (Aidenbach, Germany). Dulbecco’s phosphate buffered saline (DPBS), penicillin–streptomycin, fetal calf serum (FCS), phorbol-12-myristate-13-acetate (PMA), interferon gamma (IFNγ), lipopolysaccharides (LPS) from *E. coli*, and interleukins 4 and 13 were purchased from Sigma-Aldrich (Taufkirchen, Germany). RNeasy RNA isolation kit, Quantifast SYBR Green Master mix, RT2 First Strand Kit, RT2 SYBR Green qPCR Mastermix and RT^2^ profiler PCR Array (96-well format) human drug transporters, miRCURY LNA miRNA Custom PCR Panels, miRCURY LNA RT Kit and miRCURY LNA SYBR Green PCR Kit were from Qiagen (Heiden, Germany). The RevertAiD h Minus First Strand cDNA Synthesis Kit, the RIPA buffer, the Pierce™ BCA™ Protein-Assay-Kit, the Pierce™ ECL Western Blotting-Substrate, the anti-P-gp antibody (Clone C219, Invitrogen #MA1-26,528), and the ECL™ anti-mouse secondary IgG antibody (NXA931V) were bought from Thermo Fisher (Dreieich, Germany). The β-Actin antibody (C4, sc-47778) was purchased from Santa Cruz Biotechnology (Dallas, USA). Protease inhibitors pefabloc, leupeptin, pepstatin, and aprotinin were purchased from Carl Roth (Karlsruhe, Germany). ABSOLUTE™ QPCR SYBR® Green Mix was from ABgene (Epsom, UK). 4xLaemmli-Puffer was brought from Bio-Rad (Feldkirchen, Germany). Dithiothreitol (DDT) was purchased from AppliChem (Darmstadt, Germany). ProSieve® Quadcolor™ protein marker was purchased from Lonza (Visp, Switzerland).

### THP-1 cells’ differentiation and polarization

THP-1 cells were cultured in RPMI 1640 medium that was supplemented with 10% FCS and penicillin (100 U/mL)-streptomycin (0.1 mg/mL). Cells were constantly kept at 5% CO_2_ and 37 °C. THP-1 cells were differentiated and polarized according to our previously published protocol (Hamburg et al. 2026): 200 nM PMA for 72 h for differentiation to M0 macrophages and following a 5 days recovery period exposure for 48 h to 20 ng/mL IL-4 and IL-13 each (M2 phenotype) or 50 ng/mL LPS and 20 ng/mL IFNγ (M1 phenotype). This approach leads to correctly polarized macrophages as demonstrated by the expression of markers identifying M1 cells (tumor-necrosis factor gamma) and M2 cells (mannose-receptor C-type 1) (Hamburg et al. 2026).

### Difference of drug transporter transcriptomics between M1 and M2 macrophages

Drug transporter mRNA expression levels in THP-1 cells, differentiated M0 macrophages, or M1 and M2 cells had been evaluated previously using the RT^2^ Profiler™ PCR Array from Qiagen© (Hamburg et al. 2026). Briefly, 1.5 µg of isolated RNA (RNAeasy Kit) had been reverse-transcribed to cDNA using the RT^2^ First Strand Kit. The final PCR component mix consisted of RT^2^ SYBR Green qPCR Mastermix solution, cDNA and RNase-free water. *Beta-actin* (*ACTB*), *beta-2-microglobulin* (*B2M*), *glycerinaldehyde-3-phosphate dehydrogenase* (*GAPDH*), *hypoxanthine phosphoribosyltransferase 1* (*HPRT1*), and *ribosomal protein lateral stalk subunit P0* (*RPLP0*) had been used as reference genes. The Roche LightCycler® 480 (Roche Applied Science, Mannheim, Germany) had been used for quantifying RNA expression levels and data had been eventually evaluated via calibrator-normalized relative quantification with efficiency correction using the LightCycler® 480 software version 1.5.1.62 (Roche Applied Science, Mannheim, Germany) (Hamburg et al. 2026). The final analysis was performed using the Qiagen online tool GeneGlobe. mRNA expression of drug transporters in M1 cells was normalized to reference genes and subsequently compared to M2 macrophages that served as controls.

### P-gp protein expression evaluation

Western blot analysis was performed for P-gp in both M1 and M2 macrophages. Polarized macrophages were washed with PBS, centrifuged for 5 min at 400 g and lysed with RIPA buffer, containing protease inhibitors (1 mg/mL pefabloc, 5 μg/mL leupeptin, 1 μg/mL pepstatin, and 1 μg/mL aprotinin). Protein concentrations were measured with the Pierce BCA™ Protein-Assay-Kit according to the manufacturer’s instructions. Proteins were denaturized and diluted in 5 × SDS buffer (4xLaemmLi-Puffer + 1 × 1 M DTT) to reach concentrations of 20 µg/mL per sample.

Then, protein solutions were subjected to SDS–polyacrylamide gel electrophoresis for 120 min (stacking gel, 4%; separation gel: 10%). Proteins were subsequently blotted to a nitrocellulose membrane for 70 min and incubated in blocking buffer (5% BSA Tris-buffered saline with 0.1% Tween 20) for 30 min. Membranes were exposed to primary monoclonal antibodies over night at 4 °C (Tris-buffered saline with 0.1% Tween 20; anti-P-gp antibody diluted 1:100, anti-β-actin antibody diluted 1:2000), followed by incubation with the anti-mouse IgG horseradish peroxidase-conjugated secondary antibody (1:2000) and interim washing steps. Protein bands were stained by and Pierce™ ECL Western Blotting-Substrate exposed on radiographic film for 1–5 min. Relative P-gp expression was semi-quantitatively evaluated by densitometry (ImageJ, Bethesda, USA). Lanes from top to bottom of each sample were marked and histograms of signal intensities were recorded. Signals from P-gp were normalized to signals from β-actin.

### Evaluation of P-gp-regulating microRNA expression levels

For expressional analysis of potential P-gp-regulating microRNA species in M1 and M2 macrophages, the customized miRCURY® LNA® miRNA PCR Panel from Qiagen© was used. Those panels included primers for 46 microRNA species that had been identified to regulate P-gp expression in other cell types or tissues (Lopes-Rodrigues et al. 2014; An et al. 2017; Jiang et al. 2021; Xu et al. 2011; Kanlikilicer et al. 2018; Yang et al. 2012; Zhao et al. 2010; Ikemura et al. 2013; Zhang et al. 2018; Cui et al. 2023; Chen et al. 2012; Bourguignon et al. 2009; Asangani et al. 2008; Kim et al. 2024; Liu et al. 2021; Yang et al. 2013; Janikova et al. 2016; Feng et al. 2011; Bao et al. 2012; Shi et al. 2020; Wu et al. 2019; Gu et al., 2022; Feng et al. 2011; Xu al. 2013; Kovalchuk et al. 2008; Zhu et al. 2008; Jaiswal et al. 2012; Shang et al. 2014; Wu et al. 2016; Munoz et al. 2013; Boyerinas et al. 2012; Table S1).

M1 and M2 macrophages were obtained as described, and their mRNA was isolated using the miRNeasy Mini Kit from Qiagen©. Synthesis of cDNA was performed using the miRCURY LNA RT Kit following manufacturer’s instructions. The final PCR sample consisted of 10 µL of master mix (included in the miRCURY LNA SYBR Green PCR Kit), cDNA, and water. Four independent biological replicates per cell type were evaluated. PCR was again run in the Roche LightCycler® 480, and the final analysis of microRNA expression included the normalization to reference genes: *Small Nucleolar RNA* (*SNOR*), *C/D Box 38B*, *SNOR H/ACA box 66*, *SNOR C/D Box 48*, *SNOR C/D Box 49A*, and *SNOR C/D Box 44*.

### Statistical analysis

The statistical analysis was performed using the GraphPad Prism 9.00 software (GraphPad Software, San Diego, USA). Differences in drug transporter transcriptomics and microRNA expression between M1 and M2 macrophages were analyzed with a two-sided unpaired *t*-test. An unpaired *t*-test with Welch’s correction was performed for the western blot analysis. A *P* value < 0.01 was considered significant for drug transporter transcriptomics, while for the analysis of western blot and microRNA array analysis the significance threshold was set to *P* < 0.05.

## Results

### Drug transporter transcriptomics differences between polarized M1 and M2 macrophages

**Fig. 1:**
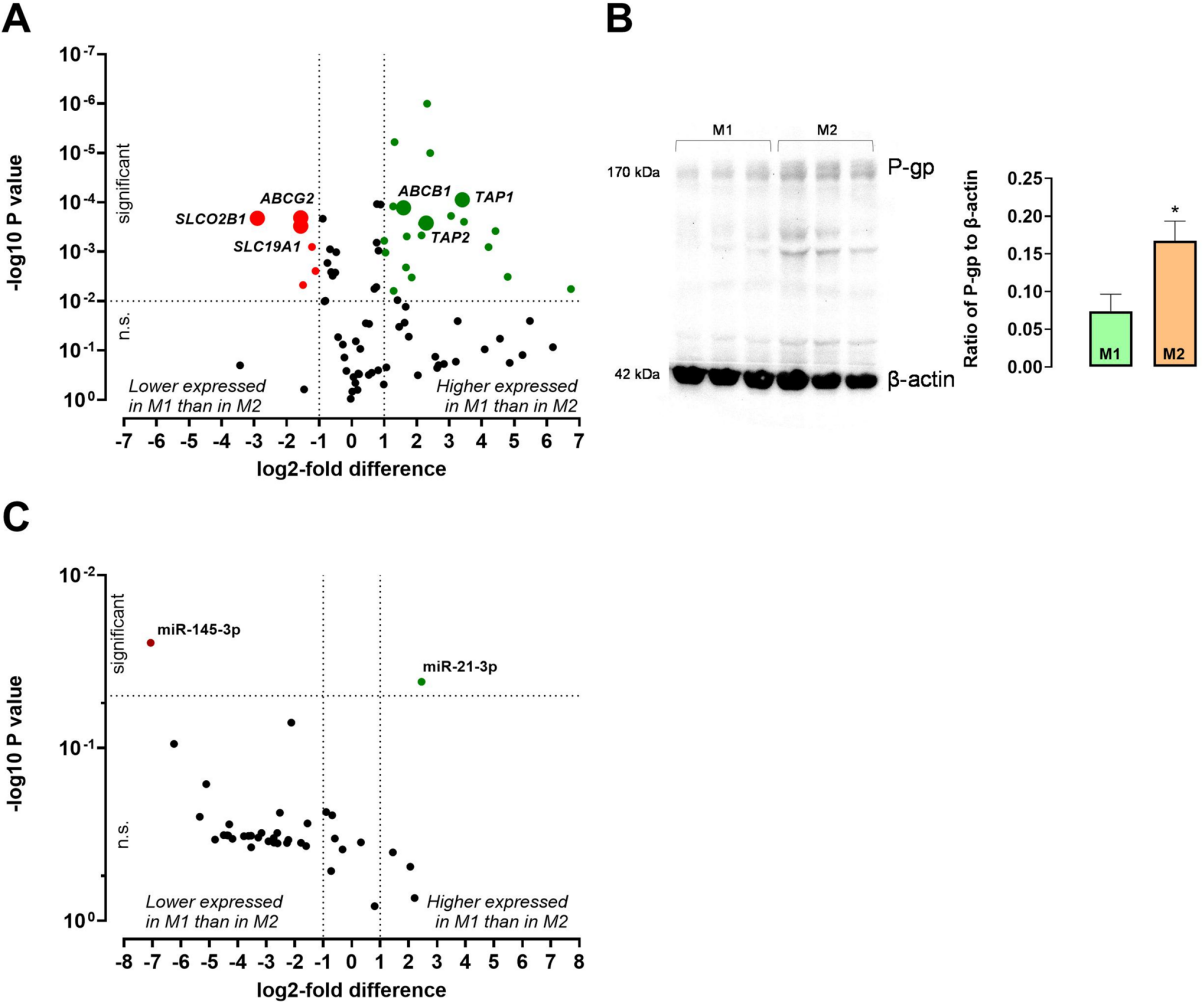
**A** Drug transporter transcriptomics in M1 macrophages, normalized to M2 macrophages. Drug transporters in M1 cells were marked as green dots if their expression was significantly (*P* < 0.05) higher at least two-fold compared to M2 cells and marked red when expression was significantly lower at least two-fold. Gene names of selected drug transporters are highlighted. Data are shown as mean ± S.D. of biological triplicates. Statistical significance was determined by parametric, unpaired two-sided *t*-test. **B** Western blot membrane showing P-gp expression (170 kDa) and β-actin (42 kDa, loading control) in M1 macrophages (three most left lanes) and M2 macrophages (three most right lanes). Semi-quantitative analysis was performed using ImageJ, normalizing signals from P-gp to signals from ß-actin. Data are shown as the mean ± S.D. of the P-gp/β-actin ratios of biological triplicates. Statistical significance was determined by parametric, unpaired t-test with Welch’ correction. **C** P-gp-regulating microRNA species transcriptomics in M1 macrophages, normalized to M2 macrophages. MicroRNA species were marked as green dots if their expression was significantly (*P* < 0.05) higher at least two-fold compared to M2 cells and marked red when expression was significantly lower at least two-fold. Names of these significantly different microRNA species are highlighted. Data are shown as mean ± S.D. of four biological replicates. Statistical significance was determined by parametric, unpaired two-sided *t*-test

To analyze differences in drug transporter transcriptomics between M1 and M2 macrophages, mRNA expression levels of 84 transporters genes were evaluated. M2 macrophages served as control for normalization. Only those drug transporter genes that were at least two-fold differently expressed with a *P* value < 0.01 were considered significantly altered. Those genes are displayed either in green (higher expression in M1 than in M2) or red (lower expression in M1 than in M2) (Fig. 1A). When compared to M2 macrophages, 20 drug transporter genes were higher expressed in M1 and seven were lower expressed. Most prominent examples for highly expressed genes in M1 cells were *ABCB1* (threefold), *Transporter Associated with Antigen Processing Proteins* (*TAP*)*−1* (10.6-fold), and *TAP-2* (4.9-fold). Genes being significantly lower expressed in M1 macrophages comprised *ABCG2* (threefold), *Solute Carrier Organic Anion Transporter Family Member 2B1* (*SLCO2B1*, 7.5-fold), and *SLC19A1* (threefold). All data are detailed in Table S2.

### Evaluation of P-gp protein expression in polarized macrophages

After mRNA analysis, determination of protein levels of P-gp was performed. The resulting Western blot is shown in Fig. 1B. Each lane represents a biological replicate of the corresponding cell type (M1 left; M2 right). The bands at 42 kDa represent the β-actin control and the bands at 170 kDa show P-gp. Densitometric analysis of the P-gp/β-actin ratio showed that P-gp is 2.4-fold higher expressed in M2 macrophages (0.17 ± 0.03; *P* < 0.05) compared to M1 macrophages (0.07 ± 0.02) (Fig. 1B).

### P-gp-regulating microRNA expression levels

M1 and M2 macrophages were evaluated for 46 potential P-gp-regulating microRNA species using PCR analysis. Only miR-21-3p was significantly higher expressed (5.5-fold) in M1 macrophages (Fig. 1C) compared to M2. On the contrary, miR-145-3p was lower expressed (128-fold) in M1 macrophages. All data are detailed in Table S3.

## Discussion

The main goal of this work was the characterization of drug transporter expression in M1 and M2 macrophages. Thus, THP-1 monocytes were differentiated and polarized to M1 cells or M2 cells using the validated and previously published protocol (Hamburg et al. 2026). While our previous work had already described changes in drug transporter transcriptomics during differentiation and polarization (Hamburg et al. 2026), we now compared the mRNA expression levels of 84 drug transporters in M1 and M2 cells for the very first time. This comparison between M1 and M2 macrophages showed that many transporter genes are differently expressed. For instance, M2 cells had more transcripts of *SLCO2B1* and *ABCG2*, which agrees with findings at the protein level (e.g., for *ABCG2*/BCRP) (He et al. 2018). However, we had an emphasis on P-gp, the ABC transporter that modulates rifampicin’s intracellular accumulation (Hamburg et al. 2026) by extruding rifampicin from macrophages (Hartkoorn et al. 2007) and affects in vitro rifampicin efficacy against Mtb (Wu et al. 2019). Its protein expression was enhanced in M2 cells, being in line with enhanced P-gp efflux activity in M2 cells (Hamburg et al. 2026). In M1 cells, most strikingly, P-gp protein expression was in fact impaired, but *ABCB1* mRNA expression was enhanced threefold in M1 cells. Therefore, an evaluation of this discrepancy was required and included the expressional analysis of 46 potential P-gp-regulating microRNA species, including those that had been verified by previous studies (see Tables S2 and S3). Their expression in M1 macrophages was normalized to the levels in M2 cells, resulting in a significantly lower level of miR-145-3p and a higher level of miR-21-3p in M1 macrophages. Both microRNA species are short, single-stranded RNA molecules that derive from their respective double-stranded microRNA precursor, which matures to 5p and 3p species (Winter et al. 2009). Both 5p and 3p species are known to co-exist and both variants are post-transcriptional regulators by binding to the 3′ untranslated region (UTR) of the respective target mRNA, hindering its translation or initiating its degradation (Bartel 2004). However, 5p and 3p variants can be functionally different, given their anti-parallel sequence (Choo et al. 2014). Sometimes, only one variant is well known, while the counterpart species is poorly characterized, or respective publications do not distinguish these two forms at all. For instance, miR-145 has been reported to down-regulate P-gp protein expression, at least in Caco-2 cells (Ikemura et al. 2013). However, when looking closely at the sequences used, one can observe that Ikemura and co-workers analyzed the 5p variant of miR-145. In contrast, our investigation constantly evaluated both variants and revealed that the 5p variant was not differently expressed between the two macrophage phenotypes, suggesting that miR-145-5p plays no major role for P-gp regulation in these cells. In contrast, the miR-145-3p variant is known to promote the M2 polarization phenotype (Huang et al. 2019) and was consequently down-regulated in M1 macrophages.

**Fig. 2:**
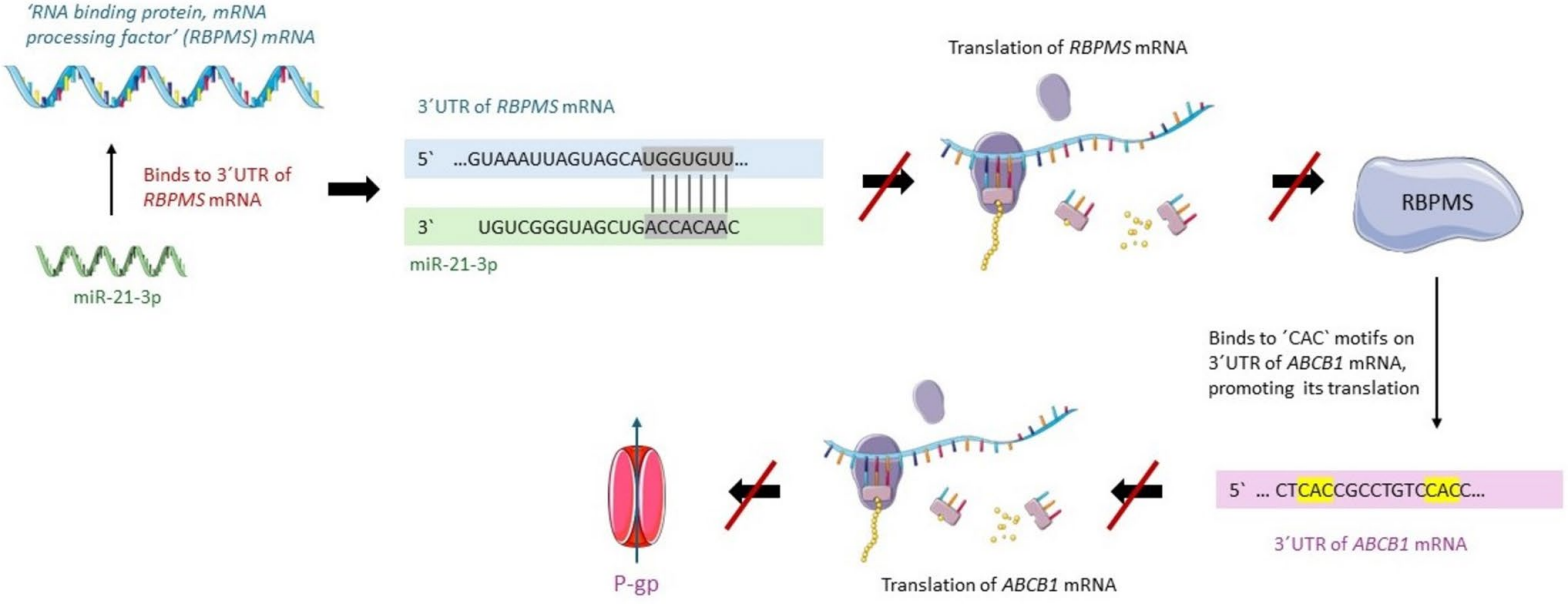
Hypothetical scheme explaining the role of miR-21-3p for P-gp regulation. miR-21-3p is a 21-base microRNA that binds to the 3′ UTR of the *RBPMS* mRNA, hindering its translation to the RBPMS protein. This protein usually binds to the CAC-rich *ABCB1* mRNA, promoting its translation to the P-gp protein. Consequently, high miR-21-3p expression is expected to dampen P-gp protein expression, while leaving high *ABCB1* mRNA levels behind as observed in M1 macrophages. The figure was created using Servier Medical Art under a CC BY 4.0 license

miR-21 is known to up-regulate P-gp protein expression, leading to enhanced cellular doxorubicin resistance (Kim et al. 2024). However, these published works again solely analyzed the 5p variant and did not investigate the impact of the 3p specie on P-gp expression (Kim et al. 2024; Asangani et al. 2008). In our analysis, miR-21-3p was significantly increased in M1 cells, which also showed increased *ABCB1* levels but low P-gp expression levels. This suggests that miR-21-3p somehow prevents the enhanced *ABCB1* transcripts from being translated to the protein. Accordingly, we searched for potential P-gp-regulating target genes for miR-21-3p using the miR database (miRBase). One such target is the “RNA binding protein, mRNA processing factor” (RBPMS). RBPMS is an important regulator of mRNA translation (Bartsch et al. 2023) and an apparent target of miR-21-3p, as evidenced by experiments performed by Báez-Vega and co-workers (Báez-Vega et al. 2016). They showed that inhibition of microRNA 21-3p enhances protein expression of RBPMS, which is known to be a co-activator of transcriptional activity (Sun et al. 2006). Therefore, the enhanced miR-21-3p levels in M1 cells should consequently reduce RBPMS, leading to low P-gp protein levels and leaving high *ABCB1* levels behind. So far, there is, however, no experimental data linking RBPMS to P-gp translation. At least theoretical considerations support the hypothesis: RBPMS binds to cytosine-adenine-cytosine (CAC) motifs of respective mRNA molecules. The sequence of *ABCB1* indeed has 17 CAC motifs in its 3′ UTR regions, while β-actin (equally expressed in both cell types) only has 7 CAC motifs. Accordingly, we suggest that RBPMS has a high affinity to the CAC-rich *ABCB1* mRNA and actually promotes its translation, unless RBPMS is inhibited by miR-21-3p (Fig. 2).

This work has its limitations. First, we evaluated drug transporter expression levels in polarized, non-infected macrophages. However, preliminary data suggest that macrophage infections (e.g., *Listeria monocytogenes*) can also have a small influence on *ABCB1*/P-gp expression (Sigal et al. 2015). Second, another weakness of this work is its descriptive nature and the missing experimental confirmation of the relevance of the identified microRNA species for P-gp expression. In contrast, we only state an indirect role of miR-21-3p in P-gp regulation. To fully prove our suggested mechanism, THP-1-derived macrophages should be transfected with miR-21-3p mimics or inhibitors and subsequently analyzed for P-gp expression or activity. However, THP-1-derived macrophages are very hard to transfect (Srivastava and Pandit, 2025; Maess et al. 2011 and Maess et al. 2014), hindering their genetic engineering. On the other hand, this work also has clear strengths. First, previous investigations only compared 45 drug transporters between monocytes and non-polarized macrophages (Moreau et al. 2011), while we compare fully polarized M1 and M2 macrophages. So, this data set for the first time reports the mRNA expression levels of 84 different drug transporters, including *ABCB1*. This confirms already published data on P-gp protein expression in M2 macrophages (Cory et al. 2016). Second, the data showed high *TAP1* and *TAP2* expression in M1 cells. Both these genes encode for “Transporter associated with antigen presentation,” a hallmark of pro-inflammatory M1 macrophages (Lankat-Buttgereit & Tampé, 2002). Consequently, *TAP1* and *TAP2* expression can be used as polarization markers, complementing classical polarization markers such as tumor-necrosis factor alpha (M1) or mannose-receptor C-type 1 (M2) (Shiratori et al. 2017). Third, this work for the first time analyzed 46 different P-gp-regulating microRNA species in polarized macrophages and put a special emphasis on evaluating both the 3p and 5p variant each, while other studies concentrated on one variant only (Kim et al. 2024 and Asangani et al. 2008). Together, our work provides insights into yet unknown differences in drug transporter transcriptomics between M1 and M2 macrophages and shows a discrepancy between *ABCB1* expression and P-gp protein expression. Subsequently, miR-21-3p is suggested to play a role in P-gp regulation in polarized macrophages.

In conclusion, M1 and M2 macrophages hugely differ by their drug transporter portfolio. M2 cells exhibit high protein expression levels of P-gp, associating with high P-gp efflux activity and diminished rifampicin uptake. In turn, M1 cells show low P-gp protein expression but high levels of its *ABCB1* mRNA and miR-21-3p, an inhibitor of RBPMS.

## Supplementary Information

Below is the link to the electronic supplementary material.

ESM1 (DOCX 31.1 KB)

## Data Availability

No datasets were generated or analysed during the current study.

## Abbreviations

P-gp: P-glycoprotein
Mtb: Mycobacterium tuberculosis
