# Supplementary material for: The striking divergence of ABCB1 mRNA expression and P-glycoprotein protein levels in M1 and M2 macrophages associates with the microRNA miR-21-3p, a regulator of “RNA binding protein, mRNA processing factor”
Source: Naunyn Schmiedebergs Arch Pharmacol. 2026 Apr 11;399(9):14601–8. doi: 10.1007/s00210-026-05288-8 (PMC13357394; doi:10.1007/s00210-026-05288-8)
Supplement: Supplementary file 1 — (DOCX 31.1 KB) [file 210_2026_5288_MOESM1_ESM.docx]

Table S1: Known P-gp-regulating microRNA species.

| microRNA | References |
| --- | --- |
| miR-107 | Jiang Let al., 2021 |
| miR-122 | Xu et al., 2011 |
| miR-1246 | Kanlikilicer et al., 2018 |
| miR-130a | Yang et al., 2012 |
| miR-138 | Zhao et al., 2010 |
| miR-145 | Ikemura, et al., 2013 |
| miR-146a | Zhang et al., 2018 |
| miR-183-5p | Cui et al., 2023 |
| miR-200c | Chen et al., 2012 |
| miR-21 | Bourguignon et al., 2009  Asangani et al., 2008  Kim et al., 2024 |
| miR-221 | Liu et al., 2021 |
| miR-223 | Yang et al., 2013 |
| miR-23b | Janikova et al., 2016 |
| miR-27a | Feng et al., 2011 |
| miR-298 | Bao et al., 2012 |
| miR-29a | Shi et al., 2020 |
| miR-302c | Wu et al., 2019 |
| miR-3178 | Gu et al., 2022 |
| miR-331-5p | Feng et al., 2011 |
| miR-381 | Xu et al., 2013 |
| miR-451 | Kovalchuk et al., 2008  Zhu H et al., 2008 |
| miR-455-3p | Jaiswal et al., 2012 |
| miR-495 | Jaiswal et al., 2012 |
| miR-508-5p | Shang et al., 2014 |
| miR-873 | Wu et al., 2016 |
| miR-9 | Munoz et al., 2013 |
| miR-let-7 | Boyerinas et al., 2012 |

Table S2: log2 fold difference in mRNA expression and corresponding P values of the tested 84 drug transporters in M1 compared to M2 macrophages. Drug transporters that are significantly different at least two-fold are marked bold.

| **Drug transporter** | **log2 fold difference** | **P value** |
| --- | --- | --- |
| ***ABCA1*** | **1.40599235967584** | **0.009526** |
| ***ABCA13*** | **2.16027483140859** | **0.000267** |
| ***ABCA4*** | **1.66902676550963** | **0.002077** |
| ***ABCA9*** | **3.05484847699562** | **0.000188** |
| ***ABCB1*** | **1.59454854955035** | **0.000128** |
| *ABCB11* | 4.55704241526372 | 0.057472 |
| *ABCB6* | 0.53605290024021 | 0.028833 |
| *ABCC1* | 0.432959407276106 | 0.027916 |
| ***ABCC10*** | **1.03562390973072** | **0.001042** |
| *ABCC11* | 1.75274859140713 | 0.052257 |
| *ABCC12* | 6.18408189037027 | 0.085286 |
| *ABCC2* | 0.176322772640463 | 0.624364 |
| *ABCC3* | 0.823749360308273 | 0.000946 |
| ***ABCD1*** | **1.6915341649192** | **0.000489** |
| *ABCD4* | 0.124328135002202 | 0.064764 |
| *ABCF1* | 0.6959938131099 | 0.005632 |
| *ABCG8* | 0.815575428862573 | 0.251105 |
| ***AQP7*** | **6.74065777162389** | **0.005676** |
| ***AQP9*** | **1.84398384404833** | **0.003306** |
| ***ATP6V0C*** | **1** | **0.000599** |
| *ATP7A* | 0.22650852980868 | 0.300101 |
| ***MVP*** | **2.41413553298445** | **0.00001** |
| *SLC10A1* | 1.62760683812965 | 0.027109 |
| *SLC10A2* | 2.83390207666916 | 0.187044 |
| *SLC15A1* | 5.48058827023386 | 0.025025 |
| ***SLC16A3*** | **1.28688114778816** | **0.006162** |
| ***SLC19A2*** | **1.31614574229336** | **0.000006** |
| ***SLC19A3*** | **4.79753183957196** | **0.003217** |
| *SLC22A1* | 3.25701061820602 | 0.025262 |
| *SLC22A2* | 4.08746284125034 | 0.094258 |
| *SLC22A3* | 2.57046293102604 | 0.13276 |
| *SLC22A6* | 5.25058266478754 | 0.123373 |
| *SLC22A7* | 2.67129337248158 | 0.197809 |
| *SLC22A8* | 0.0143552929770701 | 0.677175 |
| *SLC22A9* | 1.4594316186373 | 0.033104 |
| *SLC25A13* | 0.111031312388744 | 0.451104 |
| ***SLC28A1*** | **4.20711196120771** | **0.000804** |
| ***SLC28A2*** | **4.42088657497553** | **0.000381** |
| ***SLC28A3*** | **2.14730669878029** | **0.000464** |
| *SLC29A2* | 0.263034405833794 | 0.092203 |
| *SLC2A1* | 0.20163386116965 | 0.29125 |
| *SLC2A2* | 1.65992455840238 | 0.012987 |
| *SLC2A3* | 0.75702324650746 | 0.005148 |
| ***SLC31A1*** | **1.27500704749987** | **0.00012** |
| *SLC38A2* | 0.765534746362977 | 0.00065 |
| *SLC3A1* | 0.526068811667588 | 0.311959 |
| *SLC3A2* | 0.887525270741588 | 0.00011 |
| *SLC5A1* | 3.20006486151431 | 0.168039 |
| *SLC5A4* | 0.613531652917927 | 0.280658 |
| ***SLC7A11*** | **3.45022149589718** | **0.000247** |
| *SLC7A9* | 1.06350294230616 | 0.217968 |
| *SLCO1A2* | 2.62760683812965 | 0.225219 |
| *SLCO1B1* | 0.985500430304885 | 0.486503 |
| *SLCO1B3* | 2.03562390973072 | 0.316378 |
| *SLCO2A1* | 4.85748342828667 | 0.17703 |
| *SLCO4A1* | 0.773996325111173 | 0.000108 |
| ***TAP1*** | **3.40053792958373** | **0.000088** |
| ***TAP2*** | **2.28983446517751** | **0.000262** |
| *VDAC2* | 0.0565835283663675 | 0.342958 |
| *ABCA2* | -0.58496 | 0.003045 |
| *ABCA3* | -0.66903 | 0.000887 |
| *ABCB4* | -0.63227 | 0.002575 |
| *ABCB5* | -1.46467 | 0.609235 |
| ***ABCC4*** | **-1.46989** | **0.000207** |
| *ABCC5* | -0.42223 | 0.053486 |
| *ABCD3* | -0.74846 | 0.001687 |
| ***ABCG2*** | **-1.57046** | **0.000205** |
| ***AQP1*** | **-1.4957** | **0.004695** |
| *ATP7B* | -0.02857 | 0.947069 |
| *SLC15A2* | -3.43296 | 0.199756 |
| ***SLC16A1*** | **-1.22033** | **0.000796** |
| *SLC16A2* | -0.80735 | 0.009764 |
| ***SLC19A1*** | **-1.57046** | **0.000301** |
| *SLC29A1* | -0.88753 | 0.000214 |
| *SLC38A5* | -0.47508 | 0.001016 |
| *SLC7A5* | -0.83996 | 0.010077 |
| *SLC7A6* | -0.1635 | 0.258078 |
| *SLC7A7* | -0.50589 | 0.002625 |
| ***SLC7A8*** | **-1.11103** | **0.002443** |
| ***SLCO2B1*** | **-2.90304** | **0.000209** |
| *SLCO3A1* | -0.27501 | 0.075381 |
| *VDAC1* | -0.22651 | 0.137497 |

Table S3: log2 fold difference in microRNA expression and corresponding P values of the tested 46 P-gp-regulating microRNA species in M1 compared to M2 macrophages. microRNA species that are significantly different at least two-fold are marked bold.

| **microRNA** | **log2 fold difference** | **P value** |
| --- | --- | --- |
| hsa-miR-107 | -0.895302621 | 0.234842 |
| hsa-miR-107 | -0.687060688 | 0.245107 |
| hsa-miR-122-3p | -3.626439137 | 0.322695 |
| hsa-miR-122-5p | -2.924099886 | 0.346862 |
| hsa-miR-122b-3p | -4.501439145 | 0.319562 |
| hsa-miR-122b-5p | -4.339850003 | 0.320726 |
| hsa-miR-1246 | -3.169925001 | 0.310664 |
| hsa-miR-125a-5p | 0.321928094887362 | 0.351577 |
| hsa-miR-130a-3p | 2.06004738366994 | 0.486602 |
| hsa-miR-130a-5p | -1.782408565 | 0.35404 |
| hsa-miR-138-1-3p | -4.302319051 | 0.276875 |
| hsa-miR-138-2-3p | -4.339850003 | 0.320726 |
| hsa-miR-138-5p | 1.44360665147561 | 0.402419 |
| **hsa-miR-145-3p** | **-7.06307145** | **0.024597** |
| hsa-miR-145-5p | -1.555816155 | 0.273527 |
| hsa-miR-146a-3p | 0.807354922057604 | 0.823348 |
| hsa-miR-146a-3p | -0.722466024 | 0.515783 |
| hsa-miR-146a-5p | -1.604071324 | 0.369732 |
| hsa-miR-146a-5p | -1.608809243 | 0.369942 |
| hsa-miR-146b-3p | -0.321928095 | 0.386447 |
| hsa-miR-146b-5p | -2.72900887 | 0.353312 |
| hsa-miR-183-5p | -4.192194165 | 0.335697 |
| hsa-miR-200c-3p | -3.787641414 | 0.324395 |
| hsa-miR-200c-5p | -2.606442228 | 0.356309 |
| **hsa-miR-21-3p** | **2.4541758931858** | **0.041407** |
| hsa-miR-21-5p | -3.285402219 | 0.330426 |
| hsa-miR-221-3p | -2.121015401 | 0.071278 |
| hsa-miR-221-5p | -0.59454855 | 0.334174 |
| hsa-miR-223-3p | -2.765534746 | 0.347122 |
| hsa-miR-223-5p | -4.801676029 | 0.339058 |
| hsa-miR-27a-3p | -2.523561956 | 0.237126 |
| hsa-miR-298 | -3.533563348 | 0.375856 |
| hsa-miR-302c-3p | -4.381283373 | 0.320474 |
| hsa-miR-302c-5p | -4.339850003 | 0.320726 |
| hsa-miR-3178 | -4.339850003 | 0.320726 |
| hsa-miR-331-5p | -6.243173983 | 0.094695 |
| hsa-miR-451a | 2.21101219348551 | 0.739349 |
| hsa-miR-451b | -4.339850003 | 0.320726 |
| hsa-miR-455-3p | -2.618238656 | 0.31058 |
| hsa-miR-495-3p | -2.744161096 | 0.332455 |
| hsa-miR-495-5p | -3.538538164 | 0.322016 |
| hsa-miR-508-5p | -5.341630009 | 0.25003 |
| hsa-miR-873-3p | -4.339850003 | 0.320726 |
| hsa-miR-873-5p | -5.113950482 | 0.161714 |
| hsa-miR-9-3p | -2.22650853 | 0.340373 |
| hsa-miR-9-5p | -2.275007047 | 0.354989 |
